# Connecting with nature to mitigate toxic stress: an exploratory study of pediatric provider perceptions

**DOI:** 10.3389/fpubh.2026.1939130

**Published:** 2026-09-09

**Authors:** Candace M. Gragnani, Priyanka Fernandes, Angelica Fregoso, Florence Saez, Varsha Nair, Malakai Espinosa, Ali Al-Saleem, Connecting with Nature Community Advisory Board, Charlene Choi, Nooshin Razani

**Affiliations:** 1 Division of Medicine-Pediatrics & Preventive Medicine, Department of Medicine, UCLA Health, Los Angeles, CA, United States; 2 UCLA Fielding School of Public Health, Los Angeles, CA, United States; 3 UCLA College of Letters and Science, Los Angeles, CA, United States; 4 UCSF Center for Nature and Health, Department of Epidemiology and Biostatistics, UCSF, Oakland, CA, United States

**Keywords:** community partnership, health, nature, pediatrics, toxic stress

## Abstract

Current research supports claims for nature’s role in resilience and healing from toxic stress, adverse childhood experiences (ACEs), and their downstream sequelae. Beyond promoting individual patient health, access to greenspace has broader community-level benefits, including the fostering of community resilience and the reduction of socio-economic and race-driven health inequalities. While these benefits can dovetail with the public health goals of promoting healing, nature and nature contact remain an underutilized resource in the clinical setting with limited previous exploration as to why. The following is an overview of how nature can impact youth and families experiencing ACEs and toxic stress, with a focus on exploring insight from Los Angeles County pediatric providers and opportunities to enhance pediatric health care providers’ roles in connecting youth and families with nature to mitigate toxic stress.

## Introduction

California has committed to prevent, screen for, and address the impacts of Adverse Child Experiences (ACEs) and toxic stress. In 2019, the California Office of the Surgeon General and the Department of Health Care Services jointly launched the ACEs Aware initiative - a statewide effort to provide trainer education and support for screening, addressing, and preventing ACEs and toxic stress with the ambitious goal of cutting ACEs and toxic stress in half within a generation (1). In 2021, California passed SB 428, the ACEs Equity Act, expanding the mandate for insurance coverage for ACEs screening from Medicaid to also include commercial insurers (2).

As conversations expanded to consider effective referral pathways for positive screenings, the ACEs Aware initiative also built out resources to foster the creation of expanded trauma-informed networks of care that could combat and ideally reverse pathological toxic stress responses that increase risk for certain acute and chronic diseases across the lifespan (3–6). One such resource, the “Stress Buster” wheel, describes seven, evidence-based ways individuals can help counteract the impacts of toxic stress: (1) supportive relationships, (2) mental healthcare, (3) quality sleep, (4) balanced nutrition, (5) physical activity, (6) mindfulness practices, and (7) experiencing nature (7).

Current research supports such claims for the role of nature in resilience and healing from toxic stress, ACEs, and their downstream sequelae (8–11). In a scoping review of nature-based interventions’ impact on vulnerable youth, largely positive outcomes were seen across a variety of psychosocial and behavioral measures, particularly those around mental health, social connectedness, and personal development (12). For those living in urban areas, neighborhoods with an abundance of natural characteristics (i.e., vegetation views and bird abundance) are positively associated with lower prominence of stress, anxiety, and depression compared to neighborhoods with minimal natural characteristics (13). Access to greenspace has also been associated with improvements in attention restoration, stress moderation, stress recovery, and resilience throughout the life course (8, 14, 15), while nature contact has been found to confer additional physical health benefits such as enhanced sleep and increased physical activity (16, 17).

Although limited, the few studies examining physician recommendations to spend time in nature for health have shown high patient engagement and successful program management (18–20). As initiatives grow across the country, research on the topic of how to implement nature referrals in a clinical setting is growing (18). Early research has suggested that pediatric healthcare providers may be unaware of the benefits of nature contact recommendations and perceive their patients face barriers to accessing nature (18). Indeed, inequitable nature access impacts many communities and is more prevalent in communities with lower socioeconomic status and racial minority groups (16, 21), groups also disparately impacted by ACEs (22). A health disparity in and of itself, inequitable nature access demands health care providers play an active role in supporting and engaging patients and communities in navigating this critical determinant of health that can potentially mitigate and moderate the health impacts of toxic stress.

In Los Angeles County, where 47% of children have been reported to have experienced one or more ACEs, Black (56%) and Latinx (50%) community members are more likely to live in cities with less park space per capita and more likely to experience health disparities related to ACEs and toxic stress, such as financial hardship, higher rates of childhood obesity, and premature mortality caused by cardiovascular diseases and diabetes (23–25). Given the unique role that pediatric healthcare providers can play in fostering nature engagement through counseling and referral pathways, a community-informed exploratory evaluation of pediatric healthcare providers’ knowledge, attitudes, and beliefs about the role of nature in addressing ACEs and promoting children’s health was completed in Los Angeles County over a seven-month period from 2022 to 2023.

## Materials and methods

Utilizing a community-partnered participatory approach, the study team undertook a multiple methods and rapid community assessment approach from November 2022 through May 2023 (26). Eight key stakeholders in Los Angeles County formed the Connecting with Nature Community Advisory Board (CWN CAB). The CWN CAB was comprised of representatives from local parks and recreation, healthcare, social work, public health, community-based organizations serving families impacted by ACEs, and lived expertise from diverse and marginalized communities. The CWN CAB provided iterative feedback during study design, implementation, and data analysis. Questions for both the quantitative and qualitative portions of this study were drafted utilizing a sequential, iterative feedback method with final approval requiring agreement between the research and CWN CAB teams.

Participants were recruited through the California ACEs Aware email listserv as well as via convenience and snowball sampling through the research team’s professional networks, including the local chapter of the American Academy of Pediatrics, local health systems, and local academic medical centers. Individuals were considered eligible for participation if they: (1) were health care providers (defined as physicians, physician trainees, nurses, therapists, and/or medical assistants who worked with patients under the age of 18 years) currently practicing in the state of California; (2) were 18 years or older; and (3) were fluent in English or Spanish. Participation in either study activity did not preclude individuals from participating in the other portion of the study. All study materials and methods were reviewed and approved by the UCLA IRB-22-0901.

### Online survey

Eligible participants were invited to complete a 15-minute survey on the secure online Qualtrics platform and received $25 in electronic gift cards for survey completion. Survey respondents responded to Likert scale and open-ended questions about prior knowledge and training around ACEs, beliefs around nature’s role in pediatric health, self-report on nature referral behaviors and motivation(s) behind them, and facilitators and barriers to incorporating nature into clinical encounters and plans of care. Surveys missing more than one response were deemed incomplete and not included in the final analysis.

### Virtual focus group

Eligible participants were invited to take part in a one-hour, virtual focus group conducted on the Zoom platform and received $100 in electronic gift cards as compensation upon completion. Utilizing a semi-structured interview approach, participants responded to questions about their knowledge of ACEs, impacts of community trauma on their patients, their ideal community nature-based resource referral system, and their knowledge, attitudes, and beliefs about nature and its role in pediatric health care. After transcription of the focus group recording and review of the transcript by two qualitative research assistants not involved in focus group moderation, an inductive approach was leveraged via group discussion by the core research team to identify the major themes of the group discussion. Two research assistants then (1) jointly established a core group of salient subcodes through iterative feedback with the research team with which to better capture the essence of participant input, (2) coded the focus group transcripts independently, (3) reviewed all transcript selections for same or divergent coding, and (4) subsequently completed consensus coding to rectify any discrepant coding for all selected passages. Transcript selections were made to assign a single code to a single portion of transcript.

## Results

Thirty unique pediatric health care provider working in Los Angeles County participated in the study, with 25 survey respondents and 6 focus group participants. One participant completed both an online survey and the virtual focus group. The majority of survey respondents and focus group participants identified as white (40 and 50%, respectively) and were between the ages of 25- < 45 years (84 and 100%, respectively; see Table 1). Approximately four-fifths of survey respondents (82%) and focus group participants (83.3%) identified as female. Nurses made up most online survey respondents (44%) while all focus group participants identified as physicians (100%).

**Table 1:** Pediatric healthcare providers survey and focus group demographic data.

Demographic category	Provider online survey respondents (*N* = 25)	Provider focus group participants (*N* = 6)
	*n (%)*	*n (%)*
Age		
18 to <25 y	1 (4.0)	0 (0)
25 to <35 y	14 (56.0)	4 (66.7)
35 to <45 y	7 (28.0)	2 (33.3)
45 to <55 y	3 (12.0)	0 (0)
55 y or more	0 (0)	0 (0)
Prefer not to say	0 (0)	0 (0)
Gender		
Female	23 (82.0)	5 (83.3)
Male	1 (4.0)	1 (16.7)
Non-binary/third gender	1 (4.0)	0 (0)
Prefer not to say	0 (0)	0 (0)
Race^a^		
American Indian or Alaska Native	0 (0)	0 (0)
Asian	7 (28.0)	2 (33.3)
Black/AA	3 (12.0)	0 (0)
Native Hawaiian or Pacific Islander	0 (0)	0 (0)
White	10 (40.0)	3 (50.0)
Other	6 (24.0)	1 (16.7)
Prefer not to say	0 (0)	0 (0)
Ethnicity		
Hispanic/Latinx	6 (24.0)	0 (0)
Not Hispanic/Latinx	18 (72.0)	5 (83.3)
Prefer not to say	1 (4.0)	1 (16.7)
Occupation		
Physician (i.e., MD, DO, MBBS)	9 (36.0)	6 (100.0)
Nurse (i.e., NP, RN, LCN)	11 (44.0)	0 (0)
Ancillary health care worker (i.e., social work, administration)	5 (20.0)	0 (0)
Time spent working in health care^b^		
Less than 1 year	1 (4.0)	—
1–5 years	10 (40.0)	—
5–10 years	7 (28.0)	—
More than 10 years	7 (28.0)	—

^a^Participants could select all that apply.^b^Data only collected for online survey participants.

### Online survey

The vast majority of providers agreed that knowing about ACEs (92%) and how trauma can influence health and behavior (100%) is important for their job (see Table 2). Most (76%) survey respondents *somewhat* or *strongly agreed* that community trauma has a significant influence on the community they serve. Overall, most pediatric health care provider survey respondents *somewhat* or *strongly agreed* that nature was important for themselves (92%) and those living in the community they served (88%), with 72% who *somewhat* or *strongly agreed* that nature was easily accessible for the patients they serve. Though most (96%) *somewhat* or *strongly agreed* that knowing how nature can impact health and well-being is important for their job, 80% of respondents disclosed that they did not refer their patients to nature-based activities or local parks as part of their health care plans. In terms of receiving additional training, most health care providers agreed they would like to receive more training on ACEs (72%), trauma-informed care (92%), and nature’s impact on health and well-being (80%). Of the 25 survey participants, only 5 (20%) responded with details when asked to describe about what nature-based activities they refer patients to. Responses were activity-based, including hiking, walking, kite flying, and playing outside.

**Table 2:** Brief pediatric health care provider survey of knowledge, attitudes, and beliefs around trauma and nature in health (*N* = 25).

Statement	Strongly disagree *n (%)*	Somewhat disagree *n (%)*	Neutral *n (%)*	Somewhat agree *n (%)*	Strongly agree *n (%)*
Nature is important for my own health and well-being.	0 (0)	0 (0)	2 (8)	3 (12)	20 (80)
Nature is important for the community I serve.	1 (4)	0 (0)	2 (8)	8 (32)	14 (56)
Nature is easily accessible for the community I serve.	0 (0)	3 (12)	4 (16)	14 (56)	4 (16)
Knowing how nature can impact health and well- being are important for my job.	0 (0)	0 (0)	1 (4)	11 (44)	13 (52)
Community trauma significantly impacts the community I serve.	0 (0)	1 (4)	5 (20)	13 (52)	6 (24)
Community trauma significantly impacts the staff where I work.	0 (0)	2 (8)	9 (36)	8 (32)	6 (24)
Community trauma plays out in my workplace.	0 (0)	4 (16)	8 (32)	7 (28)	6 (24)
Knowing about ACEs is important for my job.	0 (0)	0 (0)	2 (8)	11 (44)	12 (48)
Knowing about how trauma can impact health and behavior is important for my job.	0 (0)	0 (0)	0 (0)	7 (28)	18 (72)

In response to an open-ended question about the three greatest barriers or concerns about incorporating nature into their health care plans, pediatric providers most commonly listed concerns that fell into one of three major categories: (1) lack of knowledge about safe, reliable nature-based resources available to patients; (2) belief of nature access being inequitable; and (3) fear of being perceived as dismissive by patients for recommending nature engagement that they may not readily have access to.

### Focus group

**Figure 1:**
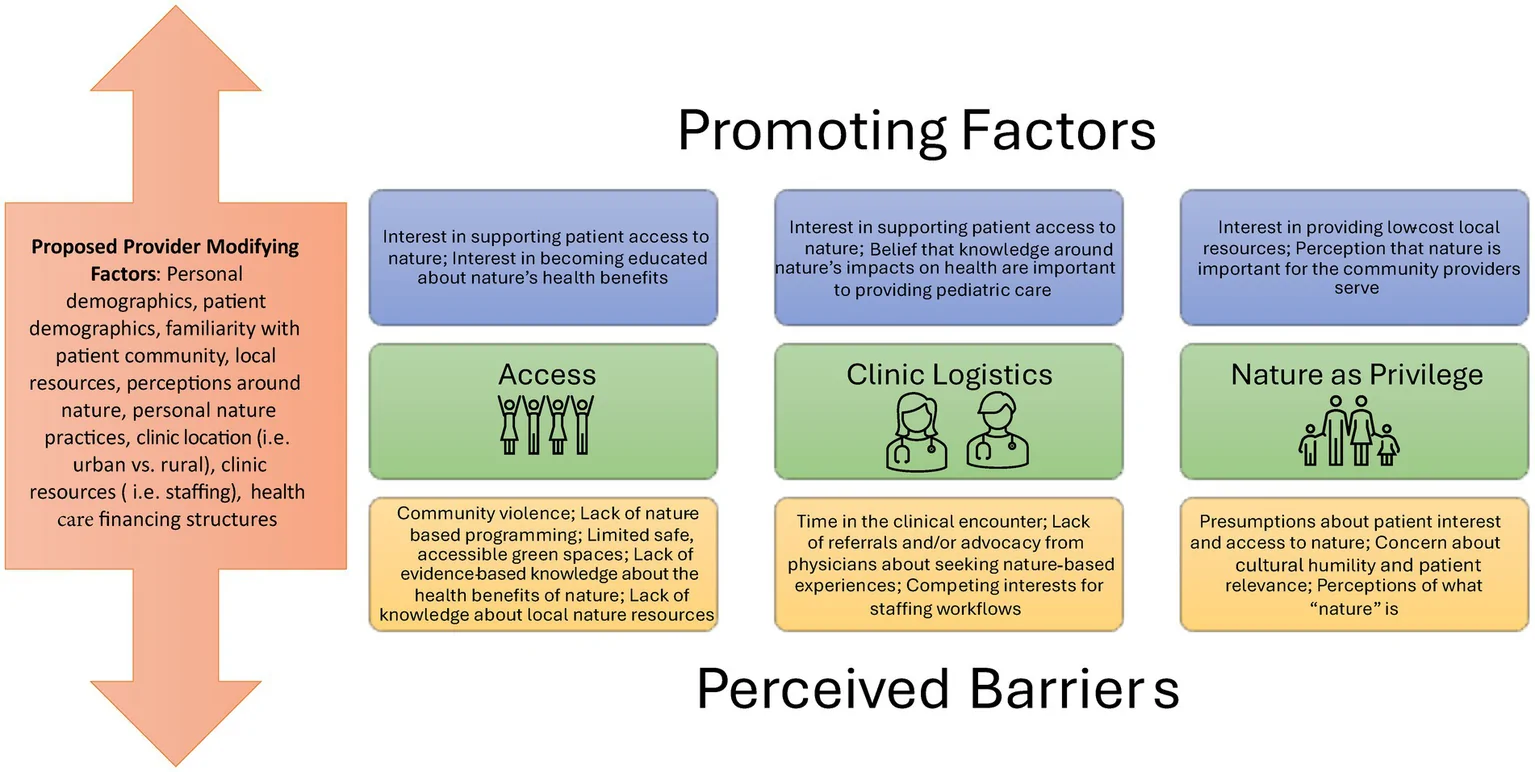
Potential modifying factors for pediatrician integration of nature-based recommendations into care.

Three core themes were identified from the discussion with six health care providers (see Figure 1).

### Access

Access remained a limitation to incorporation of nature-based interventions into care plans given (1) lack of ready access to knowledge around the evidence base for nature contact and its impacts on pediatric health outcomes; (2) lack of real or perceived local access to safe, nature-based experiences for patients and their families; and (3) lack of real or perceived access to additional resources required to engage in nature experiences (i.e., transportation, travel funding, specialized equipment). Not one of the six pediatrician participants could confidently share that they had visited a park local to their place of work and none endorsed living in the community in which they practiced.

### Nature as privilege

“Nature as privilege” was an additional critical theme that emerged from participant commentary. Providers emphasized that they prioritized considerations of context within their patients’ lives when making referrals. One participant stated that “only privileged folks can really enjoy nature,” citing a need for transportation and time off from prior responsibilities as necessities to immerse oneself in nature. Another participant added that they did not feel appropriate discussing nature or nature access in the context of additional social stressors: “It’s really about the context of the patient and where they’re at [...] trying to incorporate a nature recommendation to them if they’re like struggling with all sorts of other stressors right now.”

### Logistical considerations in clinical encounters

Providers identified lack of time during patient encounters as a key barrier to nature referrals. A participant supported this claim by stating that if they wanted to “add something else, what I’m really also saying is, ‘What am I taking away?’” It is additionally notable that discretion in the individual provider-patient encounter was noted to shape capacity for dedicated discussion and counselling around nature-based activities.

## Discussion

Children experiencing abuse and neglect are at a heightened risk of experiencing various mental and physical health-related diseases in their lifetimes, including cardiovascular disease, diabetes, substance use, and cancer (24). Nature can be an integral part of the resource path within communities that builds resilience within youth experiencing ACEs and toxic stress. This is especially true for members of underserved communities, whose health outcomes are notably and inequitably unfavorable (27). A growing body of research around nature-prescribing continues to explore the impacts of park prescriptions on objective metrics of physical and mental well-being as well as resilience (28, 29).

**Figure 2:**
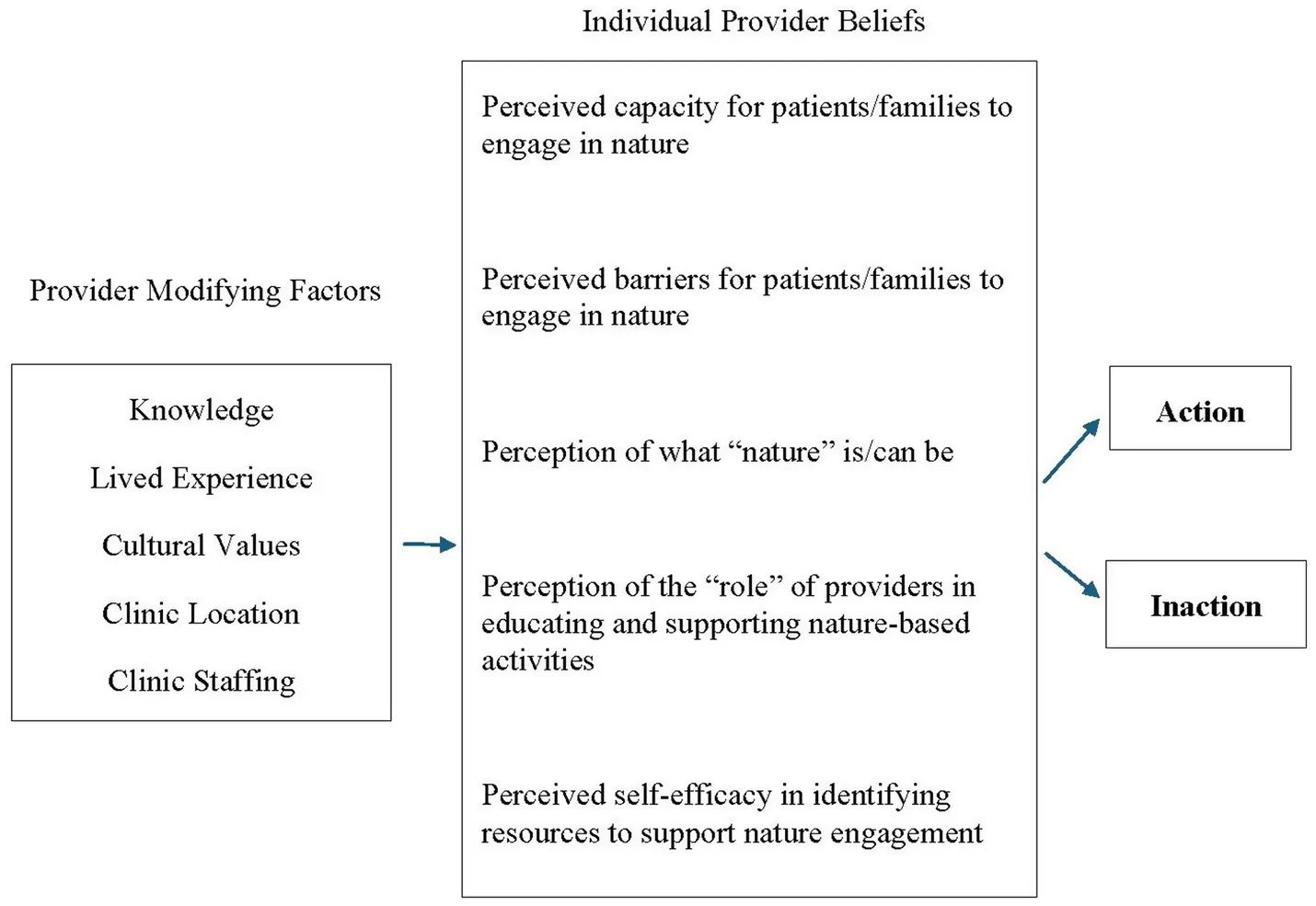
Proposed “health provider” Belief Model for integrating nature into care plans.

While this exploratory study cannot be representative of capturing generalizable knowledge, attitudes, or beliefs of pediatric health care practitioners, it nonetheless provides important insight into future considerations for more in-depth assessment and analysis. In this study, pediatric health care providers’ self-reported integration of nature into patient care was seemingly incongruent with their reported beliefs about nature’s role in health. Synthesizing quantitative and qualitative responses from this work, we propose a modified Health Belief Model – whereby health care provider perceptions influence the likelihood of provider recommendation of nature-based interventions to pediatric patients and their families (30). Further qualitative and quantitative data collection can provide additional insight into the validity of this proposed model as well as potential opportunities to expand incorporation of nature as a “Stress Buster” into health care plans and address perceived barriers to doing so (see Figure 2).

Provider participant perception of nature as privilege – likely compounded by personal and societal perceptions of what constitutes “nature” and how readily it exists in or is accessed by communities in which they work – may lead to deficit-based approaches that inhibit opportunities for patient education and engagement (Figure 2). In the era of evidence-informed decision-making, opportunities for provider education – be it formally within medical training curricula or continuing medical education opportunities – could serve to provide foundational knowledge on the research supporting nature’s role as an upstream preventive measure promoting health across the lifespan.

Nature and freely accessed communal green spaces, such as parks, are resources with great potential to foster human connection and moderate the impacts of ACEs and toxic stress across the lifespan. Evaluation of how human and capital investment on multiple levels, such as upholding the safety of communal greenspaces and fostering dynamic collaboration between community leaders and health care systems, are important areas for research on provider practice in the nature referral space.

In this limited exploration of current practices and beliefs around nature and its role in health with local providers, multiple limitations were recognized. Despite recruitment efforts spanning seven months, the small number of survey and focus group participants as well as their location in Los Angeles County limits the generalizability and comprehensiveness of responses both within and outside of the region. It is also important to note that participant demographics markedly differ from that of the Los Angeles County community at large, with a disproportionate representation of white and non-Hispanic providers compared to community demographics. Given that the focus group sample represented only pediatricians’ perspectives and did not include the input of other healthcare providers, expanded opportunities to discuss critical issues around nature-based referral systems with a range of pediatric health care providers could provide improved insight into how, where, and when such discussions and referral processes around nature-based interventions could most effectively take place.

Further research challenging the perception of nature as a privilege and examining how nature connection supports psychological well-being and physical health can strengthen healthcare providers’ ability to meet patients’ diverse and evolving needs and expand the evidence base for making such recommendations. Nature offers a unique space to build and fortify a sense of social cohesion that could be critical for the support, growth, and success of children experiencing ACEs and toxic stress. As health and public health providers, we must continue to play an active role in discovering ways to increase utilization and equitable access to nature and greenspace in our patient encounters, in our health systems, and in our communities.

## Data Availability

The raw data supporting the conclusions of this article will be made available by the authors, without undue reservation.

## Connecting with Nature Community Advisory Board

Egypt Davis-Evans, BS; Carissa Donahoo, MSW; Melanie Rojas, BA; Matthew Gonzalez, BA; Erin Higginbotham, MD; Tracey Latson; and Freddy Zavaleta, MA.
